# 4,5,6,7-Tetra­chloro-2-(4-fluoro­phen­yl)isoindoline-1,3-dione

**DOI:** 10.1107/S1600536810023032

**Published:** 2010-06-23

**Authors:** Xian-Shu Fu, Xiao-Ping Yu, Wei-Min Wang, Fang Lin

**Affiliations:** a College of Life Sciences, China Jiliang University, Hangzhou 310018, People’s Republic of China

## Abstract

The title compound, C_14_H_4_Cl_4_FNO_2_, has crystallographic twofold symmetry with the N and F atoms and two C atoms of the benzene ring located on a twofold rotation axis. The isoindole­dione ring system is almost planar [maximum atomic deviation = 0.036 (3) Å], and is twisted with respect to the florobenzene ring, making a dihedral angle of 58.56 (16)°. Weak inter­molecular C—H⋯Cl hydrogen bonding is present in the crystal structure.

## Related literature

The title compound is an inter­mediate in the synthesis of organic electro-luminescent materials, see: Han & Kay (2005 ▶). For a related structure, see: Xu *et al.* (2006 ▶).
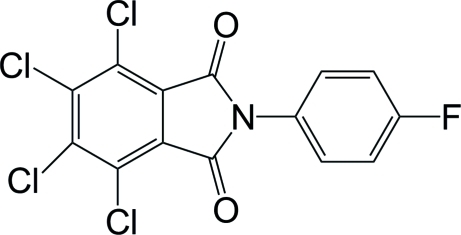


## Experimental

### 

#### Crystal data

C_14_H_4_Cl_4_FNO_2_
*M* *_r_* = 378.98
Orthorhombic, 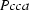

*a* = 7.9400 (16) Å
*b* = 5.6744 (11) Å
*c* = 29.461 (6) Å
*V* = 1327.4 (5) Å^3^
*Z* = 4
Mo *K*α radiation
μ = 0.91 mm^−1^
*T* = 113 K
0.20 × 0.18 × 0.12 mm

#### Data collection

Rigaku Saturn CCD area-detector diffractometer
Absorption correction: multi-scan (*CrystalClear*; Rigaku/MSC, 2001 ▶) *T*_min_ = 0.839, *T*_max_ = 0.899
6423 measured reflections
1174 independent reflections
1053 reflections with *I* > 2σ(*I*)
*R*_int_ = 0.039

#### Refinement

*R*[*F* ^2^ > 2σ(*F* ^2^)] = 0.050
*wR*(*F* ^2^) = 0.204
*S* = 1.04
1174 reflections
103 parameters
H-atom parameters constrained
Δρ_max_ = 0.84 e Å^−3^
Δρ_min_ = −0.74 e Å^−3^

### 

Data collection: *CrystalClear* (Rigaku/MSC, 2001 ▶); cell refinement: *CrystalClear*; data reduction: *CrystalStructure* (Rigaku/MSC, 2004 ▶); program(s) used to solve structure: *SHELXS97* (Sheldrick, 2008 ▶); program(s) used to refine structure: *SHELXL97* (Sheldrick, 2008 ▶); molecular graphics: *SHELXTL* (Sheldrick, 2008 ▶); software used to prepare material for publication: *SHELXL97*.

## Supplementary Material

Crystal structure: contains datablocks I, global. DOI: 10.1107/S1600536810023032/xu2780sup1.cif

Structure factors: contains datablocks I. DOI: 10.1107/S1600536810023032/xu2780Isup2.hkl

Additional supplementary materials:  crystallographic information; 3D view; checkCIF report

## Figures and Tables

**Table 1:** Hydrogen-bond geometry (Å, °)

*D*—H⋯*A*	*D*—H	H⋯*A*	*D*⋯*A*	*D*—H⋯*A*
C7—H7⋯Cl2^i^	0.95	2.80	3.690 (4)	157

Symmetry code: (i) 
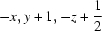
.

**Fig. 1.**
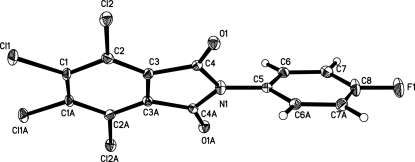
View of the molecule of showing the atom-labelling scheme. Displacement ellipsoids are drawn at the 30% probability level.

## supplementary crystallographic information

### Comment

The title compound is a key intermediate in the synthesis of organic electro-luminescent materials. The emission of light by organic molecules exposed to an electric field has been wide investigated in both an academic and industrial context (Han & Kay, 2005).

The molecular structure of the title compound is illustrated in Fig. 1. In the title compound, the dihedral angle between the benzene ring and the indole ring system is 58.56 (16)°, which is similar to 59.95 (4)° found in a related compound *N*-(2-fluorophenyl)phthalimide (Xu *et al.*, 2006). Weak intermolecular C—H···Cl hydrogen bonding is present in the crystal structure (Table 1).

### Experimental

An acetic acid solution of tetrachlorophthalic anhydride (28.6 g, 100 mmol) and 4-fluoroaniline (9.45 ml, 100 mmol) was refluxed overnight, and then filtered. The crude produce was recrystallized from ethyl acetate.

### Refinement

H atoms were positioned geometrically and refined as riding with C—H = 0.95 Å, and *U*_iso_(H) = 1.2*U*_eq_(C).

### Crystal data

**Table:**

C_14_H_4_Cl_4_FNO_2_	*F*(000) = 752
*M**_r_* = 378.98	*D*_x_ = 1.896 Mg m^−^^3^
Orthorhombic, *P**c**c**a*	Mo *K*α radiation, λ = 0.71073 Å
Hall symbol: -P 2a 2ac	Cell parameters from 3007 reflections
*a* = 7.9400 (16) Å	θ = 2.8–27.9°
*b* = 5.6744 (11) Å	µ = 0.91 mm^−^^1^
*c* = 29.461 (6) Å	*T* = 113 K
*V* = 1327.4 (5) Å^3^	Prism, colorless
*Z* = 4	0.20 × 0.18 × 0.12 mm

### Data collection

**Table:**

Rigaku Saturn CCD area-detector diffractometer	1174 independent reflections
Radiation source: rotating anode	1053 reflections with *I* > 2σ(*I*)
confocal	*R*_int_ = 0.039
Detector resolution: 7.31 pixels mm^-1^	θ_max_ = 25.0°, θ_min_ = 2.8°
ω and φ scans	*h* = −9→9
Absorption correction: multi-scan (*CrystalClear*; Rigaku/MSC, 2001)	*k* = −6→6
*T*_min_ = 0.839, *T*_max_ = 0.899	*l* = −35→30
6423 measured reflections	

### Refinement

**Table:**

Refinement on *F*^2^	Secondary atom site location: difference Fourier map
Least-squares matrix: full	Hydrogen site location: inferred from neighbouring sites
*R*[*F*^2^ > 2σ(*F*^2^)] = 0.050	H-atom parameters constrained
*wR*(*F*^2^) = 0.204	*w* = 1/[σ^2^(*F*_o_^2^) + (0.1623*P*)^2^ + 2.0637*P*] where *P* = (*F*_o_^2^ + 2*F*_c_^2^)/3
*S* = 1.03	(Δ/σ)_max_ = 0.001
1174 reflections	Δρ_max_ = 0.84 e Å^−^^3^
103 parameters	Δρ_min_ = −0.74 e Å^−^^3^
0 restraints	Extinction correction: *SHELXL97* (Sheldrick, 2008), Fc^*^=kFc[1+0.001xFc^2^λ^3^/sin(2θ)]^-1/4^
Primary atom site location: structure-invariant direct methods	Extinction coefficient: 0.039 (8)

### Special details

**Table:**

Geometry. All e.s.d.'s (except the e.s.d. in the dihedral angle between two l.s. planes) are estimated using the full covariance matrix. The cell e.s.d.'s are taken into account individually in the estimation of e.s.d.'s in distances, angles and torsion angles; correlations between e.s.d.'s in cell parameters are only used when they are defined by crystal symmetry. An approximate (isotropic) treatment of cell e.s.d.'s is used for estimating e.s.d.'s involving l.s. planes.
Refinement. Refinement of *F*^2^ against ALL reflections. The weighted *R*-factor *wR* and goodness of fit *S* are based on *F*^2^, conventional *R*-factors *R* are based on *F*, with *F* set to zero for negative *F*^2^. The threshold expression of *F*^2^ > σ(*F*^2^) is used only for calculating *R*-factors(gt) *etc*. and is not relevant to the choice of reflections for refinement. *R*-factors based on *F*^2^ are statistically about twice as large as those based on *F*, and *R*- factors based on ALL data will be even larger.

### Fractional atomic coordinates and isotropic or equivalent isotropic displacement parameters (Å^2^)

**Table:**

	*x*	*y*	*z*	*U*_iso_*/*U*_eq_	
Cl1	0.14081 (12)	0.27246 (16)	−0.00242 (3)	0.0266 (5)	
Cl2	0.01306 (11)	0.05859 (15)	0.08874 (3)	0.0246 (5)	
F1	0.2500	0.5000	0.39104 (10)	0.0445 (10)	
O1	0.0906 (3)	0.1621 (5)	0.19160 (8)	0.0236 (7)	
N1	0.2500	0.5000	0.20376 (13)	0.0224 (10)	
C1	0.1999 (4)	0.4011 (6)	0.04757 (10)	0.0199 (8)	
C2	0.1431 (4)	0.3007 (7)	0.08894 (12)	0.0209 (9)	
C3	0.1986 (4)	0.4021 (6)	0.12895 (12)	0.0184 (8)	
C4	0.1677 (4)	0.3289 (6)	0.17733 (11)	0.0183 (8)	
C5	0.2500	0.5000	0.25202 (14)	0.0173 (11)	
C6	0.1893 (4)	0.6957 (6)	0.27533 (11)	0.0206 (8)	
H6	0.1499	0.8293	0.2591	0.025*	
C7	0.1864 (5)	0.6948 (7)	0.32247 (12)	0.0264 (9)	
H7	0.1419	0.8248	0.3389	0.032*	
C8	0.2500	0.5000	0.34501 (17)	0.0338 (14)	

### Atomic displacement parameters (Å^2^)

**Table:**

	*U*^11^	*U*^22^	*U*^33^	*U*^12^	*U*^13^	*U*^23^
Cl1	0.0358 (8)	0.0274 (8)	0.0165 (7)	0.0042 (4)	−0.0031 (3)	−0.0059 (3)
Cl2	0.0301 (7)	0.0219 (8)	0.0219 (7)	−0.0043 (3)	−0.0010 (3)	−0.0042 (3)
F1	0.063 (2)	0.054 (2)	0.0166 (17)	−0.027 (2)	0.000	0.000
O1	0.0269 (14)	0.0221 (14)	0.0218 (13)	−0.0051 (10)	0.0023 (10)	0.0039 (10)
N1	0.032 (2)	0.017 (2)	0.017 (2)	−0.0042 (18)	0.000	0.000
C1	0.0228 (18)	0.0250 (18)	0.0119 (17)	0.0079 (13)	−0.0016 (11)	−0.0046 (12)
C2	0.0182 (16)	0.0226 (19)	0.022 (2)	0.0011 (13)	0.0021 (11)	0.0000 (14)
C3	0.0179 (16)	0.0204 (16)	0.0168 (17)	0.0031 (13)	−0.0014 (12)	−0.0001 (13)
C4	0.0159 (14)	0.0220 (19)	0.0171 (18)	0.0024 (14)	−0.0009 (12)	0.0013 (13)
C5	0.014 (2)	0.020 (2)	0.018 (2)	−0.0002 (18)	0.000	0.000
C6	0.0195 (16)	0.0208 (18)	0.0213 (18)	−0.0038 (13)	0.0008 (12)	−0.0016 (14)
C7	0.0243 (18)	0.030 (2)	0.0252 (19)	−0.0066 (15)	0.0024 (14)	−0.0089 (15)
C8	0.050 (3)	0.036 (3)	0.015 (2)	−0.025 (3)	0.000	0.000

### Geometric parameters (Å, °)

**Table:**

Cl1—C1	1.709 (3)	C3—C3^i^	1.379 (7)
Cl2—C2	1.719 (4)	C3—C4	1.504 (5)
F1—C8	1.356 (6)	C5—C6	1.392 (4)
O1—C4	1.203 (5)	C5—C6^i^	1.392 (4)
N1—C4	1.406 (4)	C6—C7	1.389 (5)
N1—C4^i^	1.406 (4)	C6—H6	0.9500
N1—C5	1.422 (5)	C7—C8	1.385 (5)
C1—C1^i^	1.376 (7)	C7—H7	0.9500
C1—C2	1.419 (5)	C8—C7^i^	1.385 (5)
C2—C3	1.384 (5)		
			
C4—N1—C4^i^	112.7 (4)	N1—C4—C3	105.0 (3)
C4—N1—C5	123.7 (2)	C6—C5—C6^i^	120.9 (4)
C4^i^—N1—C5	123.7 (2)	C6—C5—N1	119.6 (2)
C1^i^—C1—C2	120.7 (2)	C6^i^—C5—N1	119.6 (2)
C1^i^—C1—Cl1	120.48 (13)	C7—C6—C5	119.7 (3)
C2—C1—Cl1	118.8 (3)	C7—C6—H6	120.1
C3—C2—C1	117.6 (3)	C5—C6—H6	120.1
C3—C2—Cl2	121.8 (3)	C8—C7—C6	118.4 (4)
C1—C2—Cl2	120.6 (3)	C8—C7—H7	120.8
C3^i^—C3—C2	121.6 (2)	C6—C7—H7	120.8
C3^i^—C3—C4	108.58 (19)	F1—C8—C7^i^	118.6 (2)
C2—C3—C4	129.8 (3)	F1—C8—C7	118.6 (2)
O1—C4—N1	125.9 (3)	C7^i^—C8—C7	122.7 (5)
O1—C4—C3	129.1 (3)		
			
C1^i^—C1—C2—C3	−3.0 (6)	C2—C3—C4—O1	1.9 (6)
Cl1—C1—C2—C3	177.3 (2)	C3^i^—C3—C4—N1	3.1 (4)
C1^i^—C1—C2—Cl2	178.1 (3)	C2—C3—C4—N1	−178.2 (3)
Cl1—C1—C2—Cl2	−1.6 (4)	C4—N1—C5—C6	−122.6 (2)
C1—C2—C3—C3^i^	1.9 (6)	C4^i^—N1—C5—C6	57.4 (2)
Cl2—C2—C3—C3^i^	−179.2 (3)	C4—N1—C5—C6^i^	57.4 (2)
C1—C2—C3—C4	−176.6 (3)	C4^i^—N1—C5—C6^i^	−122.6 (2)
Cl2—C2—C3—C4	2.3 (5)	C6^i^—C5—C6—C7	−1.1 (2)
C4^i^—N1—C4—O1	178.8 (4)	N1—C5—C6—C7	178.9 (2)
C5—N1—C4—O1	−1.2 (4)	C5—C6—C7—C8	2.1 (4)
C4^i^—N1—C4—C3	−1.12 (15)	C6—C7—C8—F1	178.9 (2)
C5—N1—C4—C3	178.88 (15)	C6—C7—C8—C7^i^	−1.1 (2)
C3^i^—C3—C4—O1	−176.8 (4)		

Symmetry codes: (i) −*x*+1/2, −*y*+1, *z*.

### Hydrogen-bond geometry (Å, °)

**Table:**

*D*—H···*A*	*D*—H	H···*A*	*D*···*A*	*D*—H···*A*
C7—H7···Cl2^ii^	0.95	2.80	3.690 (4)	157

Symmetry codes: (ii) −*x*, *y*+1, −*z*+1/2.
